# Developing an Integrated Model of Care for Veterans with Alcohol Problems

**DOI:** 10.5334/ijic.5500

**Published:** 2022-02-18

**Authors:** Alison K. Osborne, Gill McGill, Peta Jane Greaves, Matthew D. Kiernan

**Affiliations:** 1 Northumbria University, UK

**Keywords:** veterans, alcohol, service provision, integrated model, healthcare

## Abstract

**Introduction:**

Veterans often do not present with alcohol problems in isolation, they may have a wide range of social, physical, and psychological needs. The aim of this study was to facilitate the development of a co-designed integrated model of care for veterans with alcohol problems.

**Methods:**

Following the development model by the Agency for Clinical Innovation, a planning symposium was held in North East of England to engage health and social care planners, public health leads, clinical commissioning groups and providers. Service users were empowered in discussions to provide insights and look for solutions (N = 43).

**Results:**

Using diagramming techniques, three examples of health and social care provision were created demonstrating the current commissioning landscape, one veteran’s experience of accessing services and a proposal for a new integrated model of care for veterans with alcohol problems.

**Discussion:**

By engaging stakeholders and service users, the model proposed a potential solution to reducing the number of veterans ‘falling through the gaps’ or disengaging from services. The collaborative approach highlighted the difficulties in navigating the current complex health and social care systems. The co-designed hub and spoke model aims to enable alcohol misuse services to adapt and evolve so that they better fit the needs of veterans.

## Introduction

It is estimated that 2.5 million UK Armed Forces veterans reside in Great Britain [1]. However, the way in which veterans are defined varies across the world, from very specific to more broad definitions [2]. In the US and Canada, to be defined as a veteran, you must have been discharged or released under conditions other than dishonourable. Australia define veterans more broadly as someone who has “rendered eligible war service”, or “is a member of the defence forces”. The UK has the most inclusive definition where a veteran is anyone who has “served for at least a day in Her Majesty’s Armed Forces, whether as a Regular or as a Reservist” [3]. It is important to consider how veterans are defined as this underpins their eligibility to access specific health and social care service provision.

Military service can have a long-term impact, across multiple life domains and social relationships, is contingent upon the social and historical context of service and can manifest in potentially both positive and negative outcomes [4]. Although most service leavers transition to civilian life successfully, research has shown that, there are higher rates of homelessness, alcohol misuse, domestic violence, relationship breakdown and criminality amongst former military personnel with untreated mental health problems [5,6,7,8]. Specifically, Iversen, Dyson (9) found that in a sample of 496 veterans, 11.8% were alcohol dependent. Additionally, Buckman, Forbes (10) found the prevalence of alcohol misuse was higher among a sample of 845 veterans (16.8%) than among the general population (4.7%) regardless of length of service.

There is a general consensus that veterans present with a wide range of social, physical and psychological needs caused by or contributing to their alcohol problems [11,12,13]. Military experience itself can influence the behaviour of veterans, shaping how they respond to challenges in civilian life [14]. In this regard, it is essential that health providers understand the characteristics of the veteran population and are aware of the cultural sensitivities associated with having been a member of the Military, to ensure the provision of clinically appropriate care [15]. The US, Canada and Australia all have specific departments dedicated to providing or funding healthcare for veterans in addition to their eligibility to access general health care systems (including private sector). The UK does not have the same structures in place, instead veterans access healthcare through the National Health Service like the general population, with further support from the charity sector.

With regard to substance misuse services, there is a growing body of research highlighting the potential benefits of integrated care as a way of addressing the needs of people with alcohol problems, given the broad range of other issues often also experienced [16]. However, services have not always been equipped to address the multiple and complex needs that people with alcohol issues present with, resulting in the development of separate services with no clear pathways. In addition, services have not addressed or been able to engage service users in the development of services. As a result of alcohol misuse being accepted widely as an ongoing issue within the veteran communities worldwide, recent research has examined the efficacy of treatment models [17,18,19,20]. For example, treatment has historically fallen into two categories, serial treatment and simultaneous/parallel care provided within existing treatment programmes and settings. The multiple, complex needs experienced by veterans with alcohol issues often requires access to support from both mental health and substance misuse services. This involves delivery by mental health clinicians for mental health treatment with treatment for substance misuse issues provided by addiction treatment clinicians. The problem associated with this model is that co-ordination between the two is variable. The UK Health and Social Care Act (2012) renders substance misuse services the responsibility of local government within public health and social care, completely separate to healthcare provided through the National Health Service [21].

The then Minister for Health (2010–2012), Andrew Lansley, said the UK Health and Social Care Act had three key principles: patients were to be at the centre of the National Health Service; a change in the emphasis of measurement to clinical outcomes; and empowering health professionals, in particular General Practitioners (GP, Family Doctors in Primary Health). With estimates of a £30 billion funding gap by 2020, a review of the way health services are currently delivered remains high on the policy agenda. The Five Year Forward View has stressed that developing innovative approaches to delivering healthcare are integral to the long-term future of the National Health Service. Creating a model of care in partnership with service users aligns with the aims of the National Health Service ‘Five Year Forward View Plans’ [22].

In the UK, National Health Service providers are required to have a set of common access policies to ensure equity of access for veterans and their families. National Health Service England expect providers to have a due regard to the Armed Forces Covenant [3] in managing their waiting lists and inter-provider transfers. The Covenant outlines that veterans and their families should not encounter disadvantage as a result of their service and that, where appropriate, special consideration should be given [3]. Despite this expectation, recent studies show that health, social and welfare provision for veterans remains inconsistent and fragmented with poor communication between agencies in the planning and delivery of care [11,23]. To address this, it is argued that health and social care organisations need to adopt a collaborative framework in the planning and commissioning of delivery of care for veterans with specific Armed Forces service-related difficulties. The UK government recognises that many organisations, both government and charity, can be involved in delivering care to veterans, and coordinating this delivery can be problematic. Within the UK government’s Veteran Strategy, two of the cross-cutting factors specifically aim to improve the collaborative delivery of specialist veteran care between the state and the charity sector, with a move to improved coordination of these services [24]. The aim of this study is to facilitate the design of an integrated model of care to enable alcohol misuse services to adapt and evolve so that they better fit the needs of veterans.

## Methods

### Summary of Phases 1–3

This study was undertaken as the last phase of a larger four-phase study looking to understand the complexities veterans experience in accessing substance misuse care [26 for full project report].

The aim of Phase 1 of the study was to investigate the perceived barriers to care amongst those planning, commissioning, and delivering services for veterans with substance misuse problems. This phase of the study found that complexity of services and care, complexity of need and a lack of understanding of military and veteran culture and the nature of modern warfare were identified as factors that made accessing substance misuse care difficult [11].

Phase 2 explored why veterans in the UK were either reluctant or had difficulty in accessing help for alcohol problems from a service users’ perspective. This phase of the study found that veterans appeared to excuse or normalise their excessive alcohol consumption, which led to a delay in meaningful engagement in substance misuse services, resulting in complex and complicated presentations to health and social care services. The findings of this phase clearly suggested that veterans who misuse alcohol have a range of distinctive and unique difficulties that subtly differentiate them from the wider civilian substance misuse population, and that the use of peer-support models would appear to mitigate against veterans disengaging from alcohol treatment services [25].

Phase 3 focused on veterans from the wider community, who themselves did not have significant problems with alcohol misuse, to determine the wider beliefs around substance misuse within the military community. The findings of phase 3 reinforce the findings of phases 1 and 2, in that there was a perceived belief that military culture conditioned veterans (and service personnel) to be resilient, avoid help seeking behaviour, view injury and illness as a weakness and encouraged alcohol use as a coping mechanism. When reflecting upon these beliefs, it reinforced the findings of the culture identified in phases 1 and 2 and added a depth of understanding as to why the participants in phase 2 not only viewed their alcohol consumption as acceptable and ‘normal’, but were also, potentially, very proud of the extent to which they could drink [26].

Findings from the first three phases were used to provide participants with an evidence base for the difficulties that veterans experienced in accessing and engaging with alcohol services in their locality. The participants who took part in the first three phases, were invited to contribute to phase four, the planning symposium, to collaborate in the development of an integrated model.

### Design and Setting

To achieve the aims of phase 4 of the study, a one-day planning symposium was held, with the aim to develop a co-designed integrated model of care for veterans with alcohol problems. Designing an integrated model of care is complex and to aid the development, the design of the planning symposium was based on the development model by the Agency for Clinical Innovation [27]. This was chosen for the importance placed on first defining the problem, understanding the ‘as is’ and then developing the ideal model. Furthermore, the guiding principles of the development of a Model of Care aligned with the aim of the current study. The principles included: a focus on empowering service users by being patient centric, supporting integrated care, supporting efficient utilisation of resources, supporting safe, quality care for patients, is innovative and considers new ways of organising and delivering care and sets the vision for services in the future.

**Figure 1:**
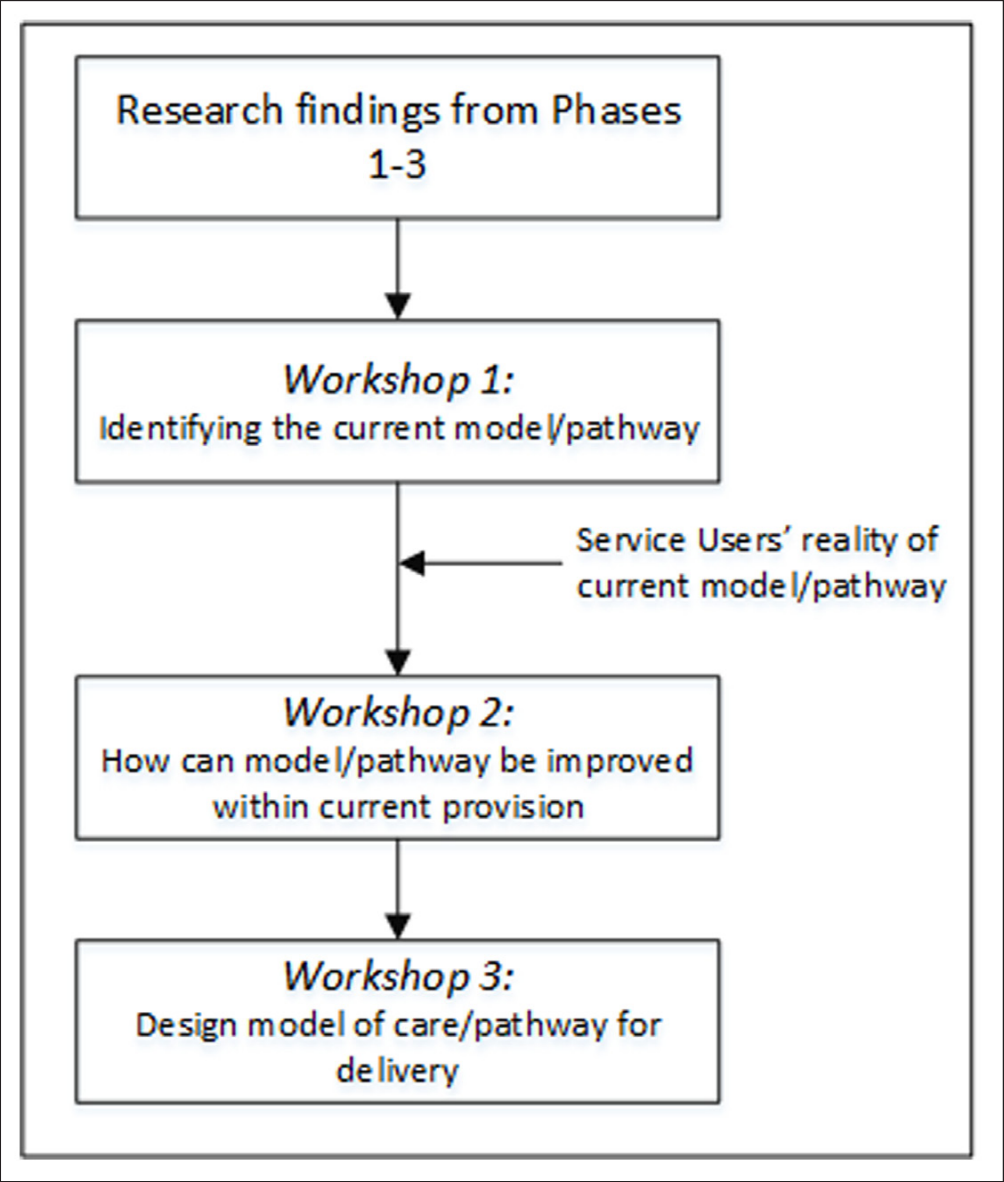
Planning Symposium process.

After presenting the findings from the first three phases of the study, participants took part in three 1-hour workshops (see ***Figure 1***). The aim of the workshops were:

1. To map the current substance misuse care pathways for veterans within their area.
2. To explore how care delivery could be improved within current provision.
3. To co-design a model of care delivery for veterans with substance misuse.

### Participants

Invitations to participate were sent to 148 health and social care planners, public health leads, clinical commissioning groups and service providers who were involved in alcohol and substance misuse provision in the North East of England, with 29% (n = 43) consenting to take part in the planning symposium. Participants were split into four groups (eight participants in each) representative of the geographical area in which they worked (where applicable). In addition, there was an ‘expert’ group (six participants who had knowledge of the National Health Service, criminal justice, public health and charity sector) and an Armed Forces veteran group which consisted of five service users and members of the wider veteran community in the North East of England. This group and the expert group were available for the area groups to call on for experience and insight.

### Data Collection

Each aim formed the basis for each workshop, encouraging participants to visualise care pathways to be recorded by facilitators. These workshops were designed to allow participants to discuss insights and solutions, not always available through traditional approaches. This approach is similar to the use of focus groups for planning, goal setting and needs assessment [28]. Facilitators of workshops were academics with current/prior clinical experience in the National Health Service and used Lean Six Sigma principles to encourage participants to examine pathways [29]. This approach revealed problems such as unnecessary delays, unnecessary steps, bottlenecks, or barriers that may contribute to veterans abandoning the system and has been used to improve patient services [30,31]. Participants generated data through the development of a series of diagrams from each workshop. The agreed diagrams detailed relationships and pathways of care that illustrated their understanding of the links between the services that they were describing within their own localities. Diagramming techniques are extensively used to display quantitative data but can also be of value in interpreting qualitative data [32].

Following each workshop, participants from each area group provided feedback to the wider symposium on their diagram of the current pathways and suggestions for improvement. This ensured all participants were provided with the same information to allow them to propose an integrated model of care more effectively. Thus, workshops one and two prepared the participants for the final workshop. In the final workshop, participants were tasked to develop an integrated model of care for veterans with alcohol problems. There were two constraints they were required to adhere to: (1) any model of care needed to be delivered within current budgets, (2) no development of parallel, bespoke services were allowed. Central to the planning symposium was the co-production of a model of care by health and social care planners, public health leads, clinical commissioning groups, providers and service users based on the empirical evidence provided by the research team and evidence provided by the wider symposium.

Facilitators transcribed the diagrams given by the participants from the workshops on to flipcharts and a diagram from each workshop was created in Microsoft Visio, representing all groups. No further analysis was undertaken. By voluntarily agreeing to take part in the planning symposium, participants gave their informed consent – this was discussed with participants at the beginning of the study. This study was granted ethical approval through Northumbria University’s Ethical Approval System.

## Results

**Figure 2:**
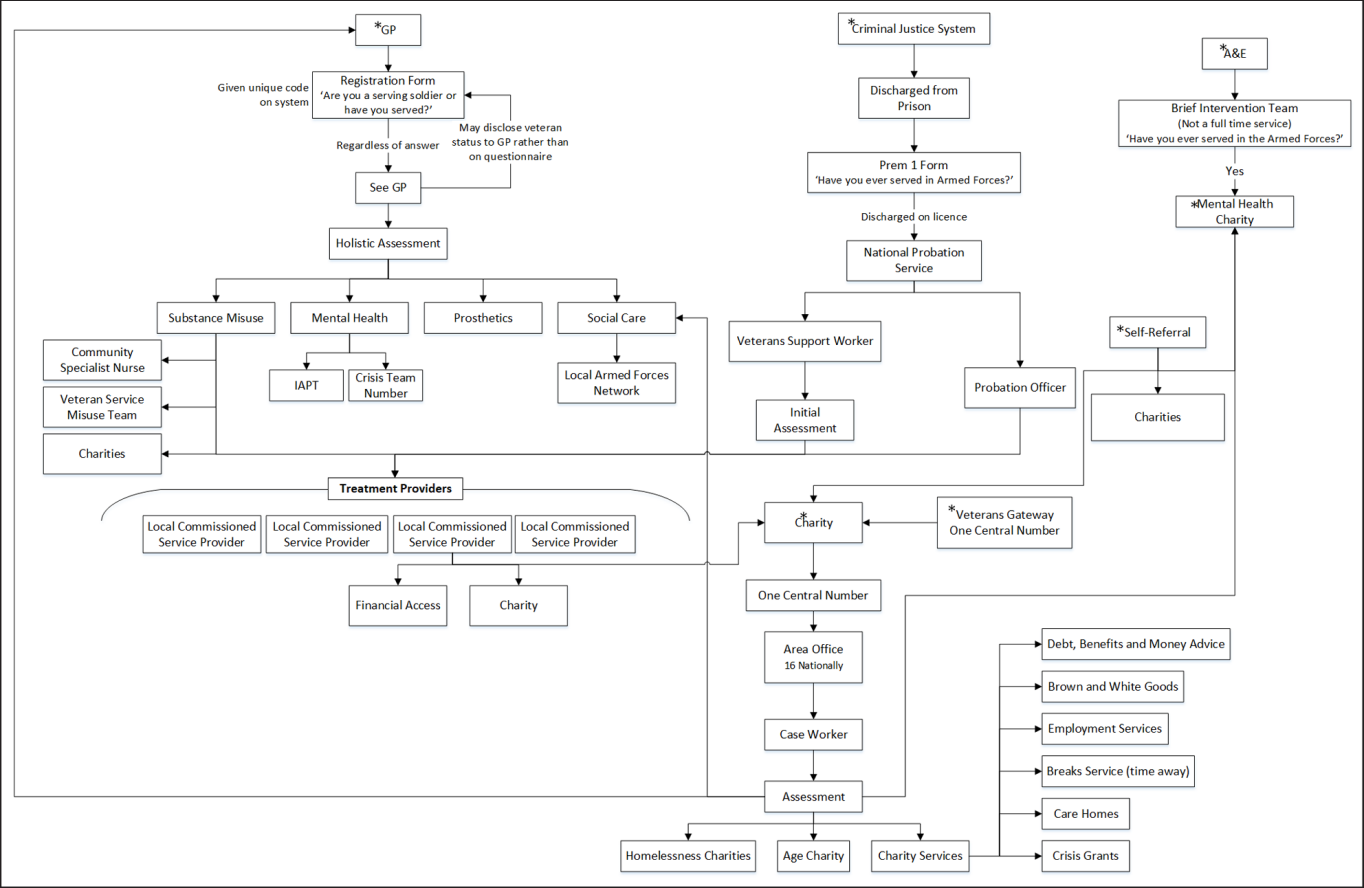
Collated diagram for Existing Landscape: Current Commissioning of Services for Veterans. *Note*: IAPT - Improving Access to Psychological Therapies. *Represents potential entry points into accessing support.

Data collected at the workshop was collated to create three overall diagrams that most accurately displayed the findings from the day regarding current provision and how to develop a co-designed, integrated service provision:

- Existing Landscape: Current Commissioning of Services for Veterans (***Figure 2***)
- Veteran’s Experience of Accessing Services (***Figure 4***)
- Improving Care and Care Pathways: Forward View Plans (***Figure 5***)

### Existing Landscape: Current Commissioning of Services for Veterans

**Figure 3:**
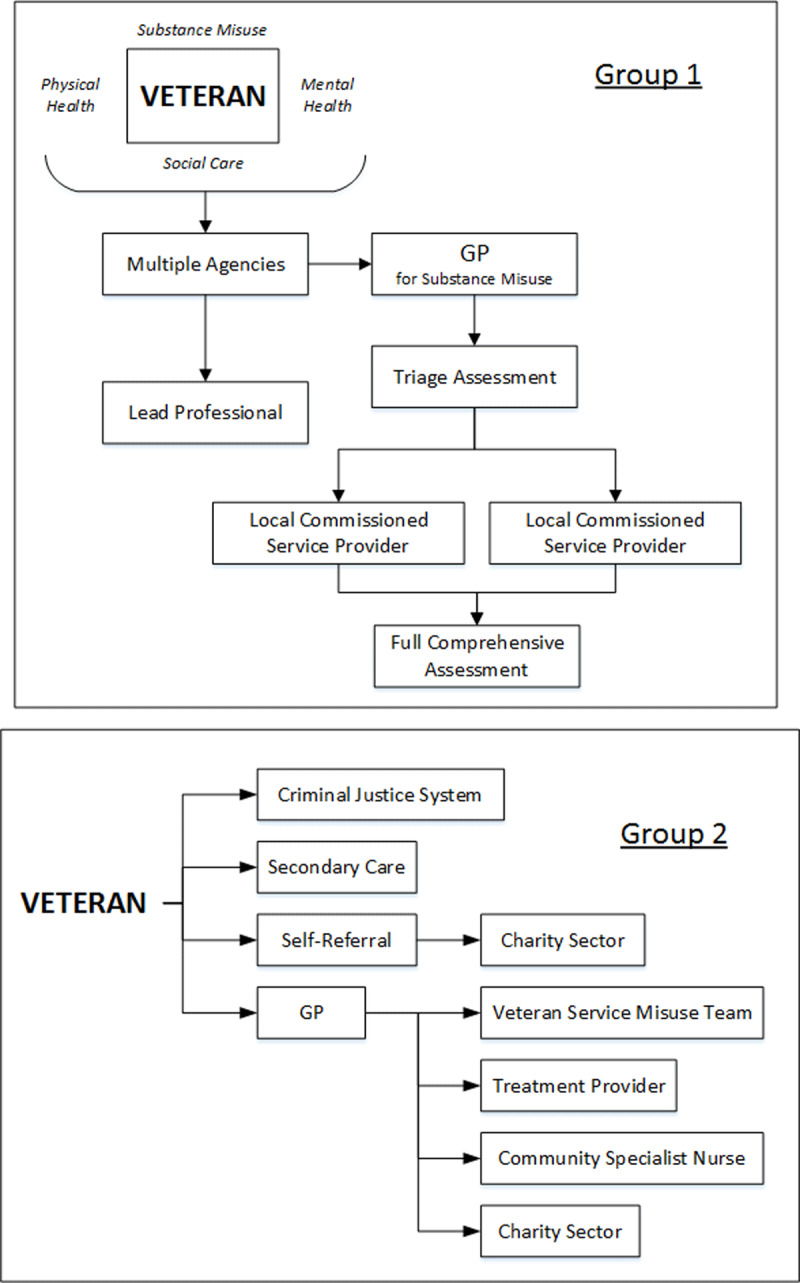
Example diagrams from Groups 1 and 2 in Existing Landscape: Current Commissioning of Services for Veterans Workshop before collation (figure 2).

***Figure 2*** represents the collation of findings from workshop one, where participants mapped the current provision. Initial diagrams from each area group presented a very simple pathway for veterans accessing healthcare for alcohol problems (see ***Figure 3*** for example). When this data was collated, it became evident that existing pathways into services were elaborate and complicated. Following data collection, facilitators helped service commissioners, planners and providers to explore their over-simplified view of the current provision within their respective localities. ***Figure 2*** represents an example of current healthcare pathways for veterans with alcohol problems as identified by the participants. There was an acknowledgment from health and social care planners, public health leads, clinical commissioning groups, service providers, service users and academics that workshop one highlighted the need to challenge their current thinking and understanding of service delivery and access to provision.

***Figure 2*** represents an example of the healthcare pathways for veterans with alcohol issues. This example was designed from the data collected and collated from participants during the workshop (planners, commissioners, and service providers) to demonstrate the diversity of treatment providers commissioned across the region. It also aims to highlight multiple entry points for veterans accessing help, with repeated referrals to a General Practitioner and National Health Service treatment providers from other organisations and referrals to charities. Participants suggested that the reason for this may be due to the veterans presenting with other problems they require support for, in addition to their substance misuse problems, or they found veteran-specific third sector charities more person-centred and sensitive to their needs.

### Veteran’s Experience of Accessing Services for Veterans

**Figure 4:**
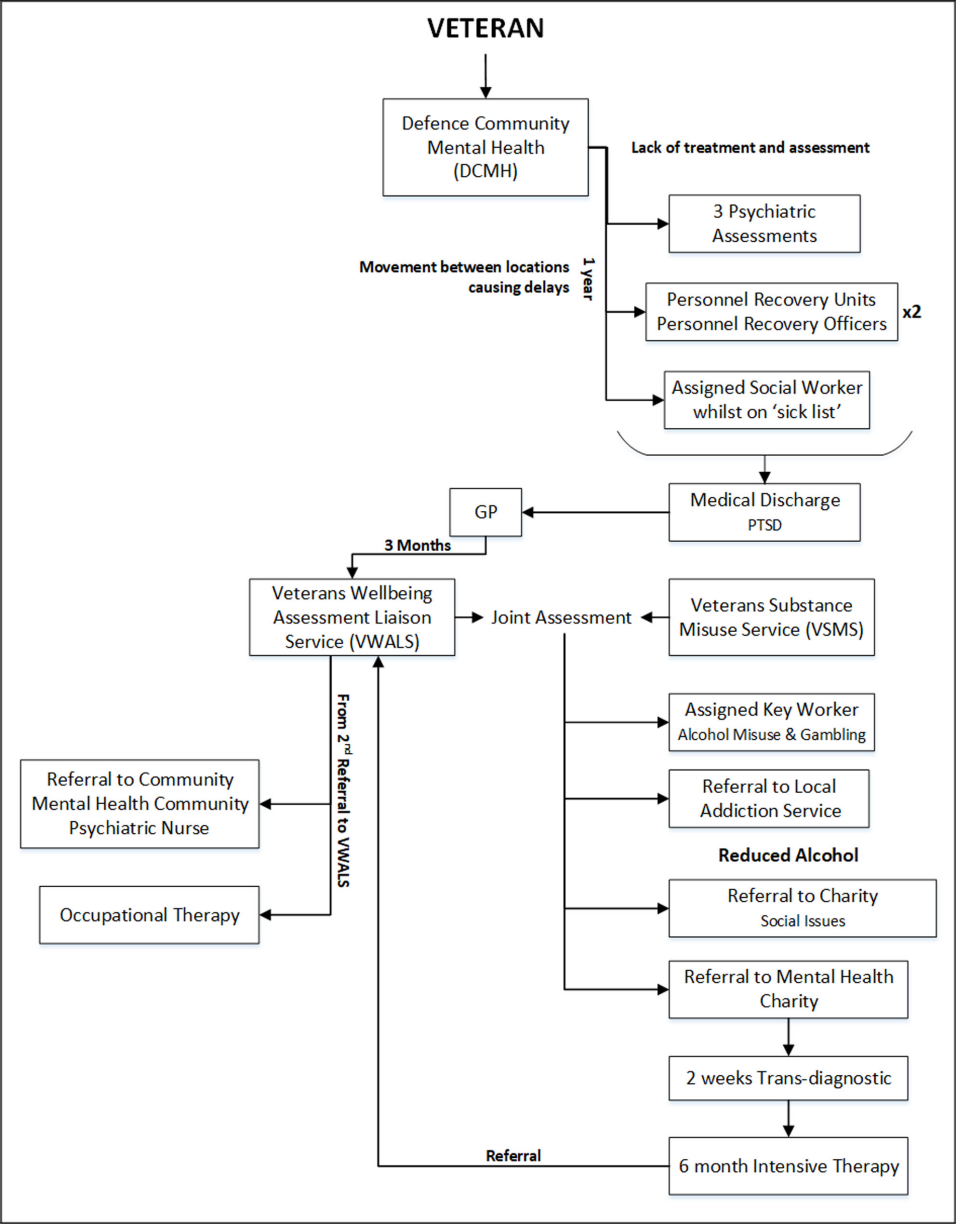
An example of a Veteran’s Experience of Accessing Services. *Note*: VWALS was the veteran specific mental health service.

It was important for participants to understand veterans’ experiences as service users to appropriately consider areas for improvement within current health and social care pathways. Following workshop one, service users (from the Armed Forces veterans group), were encouraged to provide their personal experience of accessing help for alcohol problems. As a result, there was a significant variation in experience of looking for support. For some, attempting to navigate services alone was extremely difficult. For example, veterans reported multiple re-referrals and little or no contact from service providers following assessment. The veteran’s experience, presented in ***Figure 4***, was the most comprehensive model of care discussed in the Symposium and was thought to be the best representation of a veteran’s experience. The veteran in ***Figure 4*** initially started their care pathway whilst serving in the UK Armed Forces; consequently, their experience of service pathways within the existing model of care (***Figure 2***) was more comprehensive than some others in the service users and veterans’ group.

The veteran, in ***Figure 4***, initially accessed support through Defence Community Mental Health, a mental health service for serving personnel. On discharge from the Armed Forces, the veteran was referred to a General Practitioner. From this point, ***Figure 4*** demonstrates the complex realities of the care pathway represented in ***Figure 2***.

The General Practitioner referred the veteran to a National Health Service veteran specific mental health service and a veteran specific substance misuse service where they received a joint assessment. This led to multiple referrals to secondary care and charity sector organisations before a re-referral back to the veteran specific mental health service. For the veterans this was identified as a very confusing pathway that was difficult to navigate. There were a number of agencies involved and no explanation was provided for multiple assessments and referrals. For the service providers in particular, this confusion described by the veterans was unexpected and different from their over-simplified view of existing provision (see ***Figure 3***).

***Figure 4*** identifies multiple points where the veteran could have ‘fallen through the gaps’ in care. Participants identified that service users appeared to become stuck in a cyclic, or ‘revolving door’ relationship with the substance misuse services, consisting of admission to care, disengagement, deterioration and then readmission. How services communicated with each other remained unclear, however, what was both interesting and perplexing was that specialist services that the National Health Service veteran specific mental health service had referred the veteran to for treatment, were subsequently referring the veterans back to the National Health Service veteran specific mental health service. The National Health Service veteran specific mental health service was an assessment, peer support and signposting service, which ensured veterans were engaged with services they required. Therefore, it is difficult to understand why service users whose needs had been identified, were being sent back to the National Health Service veteran specific mental health service from the service providers they had been sent to for treatment.

### Improving Care and Care Pathways: Forward View Plans

Taking on board the evidence from the first three phases (***Table 1***) and the initial workshops, there was a consensus that navigating the current model of care through complex linear pathways (***Figure 2***) was ineffective in engaging and retaining veterans in services. Health and social care planners, public health leads, clinical commissioning groups, service providers and service users started to define and describe an improved model of care. As a result, the service users and the wider participants of this study designed and recommended a ‘hub and spoke’ model of care delivery. This was a conscious move away from a linear pathway of care, as participants argued that such model would increase the risk of disengagement when service users moved from one service to the next. The participants felt that a hub and spoke model would be the most effective way of integrating health and social care delivery for optimal future healthcare services.

**Table 1:** An overview of the first three phases from the wider study [26].


	DATA COLLECTION	PARTICIPANTS	KEY FINDINGS

**Phase One**	Semi-structured interviews	Health and social care planners, commissioners and providers	Complexity of servicesand care Complexity of need Lack of understanding of theArmed Forces culture

**Phase Two**	Semi-structured interviews	Armed Forces veterans service users	Normalisation of alcohol consumption Delay in meaningful engagement Complex presentations Benefit of peer-support models of care

**Phase Three**	Focus group	Veterans from the wider community	Veterans (and military personnel) conditioned to be resilient Poor help-seeking behaviour Armed Forces cultural considerations


**Figure 5:**
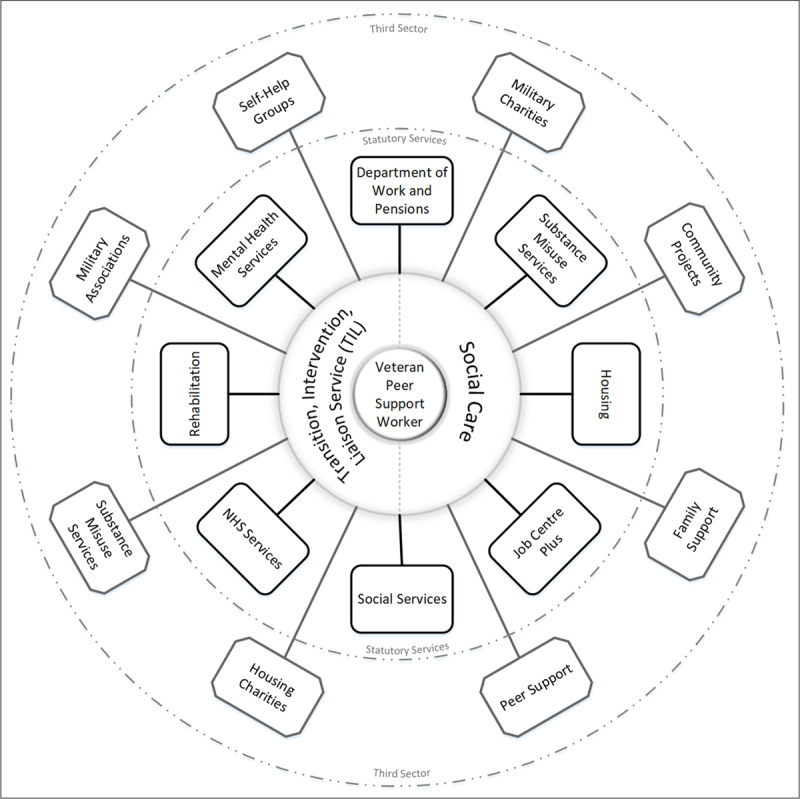
Hub and spoke model for Improving Care and Care Pathways: Forward View Plans. *Note*: Transition, Intervention and Liaison Service is the Veterans’ Mental Health Service.

In line with the constraints set out at the start of the symposium, it was argued that the proposed model could hypothetically be implemented without the need for physically integrating health and social care budgets. Any model that relied on integration of budgets would arguably make the process too complicated to succeed. A ‘Veterans’ Hub’ (see ***Figure 5***) was placed at the centre of this model, where veteran peer support workers would be integrated with the National Health Service veteran specific mental health service.

In practice the model would see veterans who require mental health or substance misuse services at the centre, supported by a veteran peer-support worker. The hub (***Figure 5***) would be physically located within local National Health Service veteran specific mental health service. The veteran peer-support worker would look to empower the veteran and maintain contact throughout their treatment journey. To support the implementation of this model in practice, it is recommended that the peer-support worker is recruited from the existing workforce (e.g., veteran specific mental health service) with knowledge of local services. They will act as the link between all services for the service user and most importantly will seek to reduce the risk of the service user disengaging from care. It is envisaged that even if a veteran disengages from treatment, they would not disengage from the hub, and the role of the veteran support worker would then focus on re-engagement. It was argued that this model would prevent veterans becoming lost in the system as outlined in ***Figures 2*** and ***4***.

The strength of this hub and spoke model is that it places responsibility of the peer-support worker with a single organisation without compromising the need to take a holistic approach to the health and social care needs of veterans affected by substance misuse. In addition, competing budgets can make it difficult to negotiate care provision, this co-designed model is cost-neutral and potentially provides a solution to removing barriers that exist across the health and social care sectors.

## Discussion

The planning symposium identified variable pathways currently in place for veterans accessing help for alcohol problems, particularly in terms of complexity and potential confusion. For service users, there were multiple entry points with the potential to ‘fall through the gaps’. Despite this and previous research identifying that health and social care provision is complex and difficult to navigate, this study’s sample of service planners, commissioners and providers had an over simplified view of existing provision. Participants were ‘surprised’ service users typically experienced confusion, delays and multiple assessments and referrals, each one increasing the likelihood of disengagement from services and a future reluctance to engage. The planning symposium provided service users the opportunity to share their experience of the current model of care for veterans accessing help for alcohol problems. This was highly advantageous to the wider participants, who acknowledged that planning symposium had highlighted the complexity of current provision that were previously unaware of. Consequently, participants were able to start to envisage and plan a new co-designed integrated model of care.

Some of the key aspects of the current care pathway that required improvement were discussed from a ‘whole systems perspective’. Participants cited key reasons for a lack of effective engagement of veterans in services including: a lack of understanding of veterans and the term ‘veteran’ itself, complexity of services, a normalisation of alcohol consumption and complex case presentations [e.g. 11,25,33,34,35]. In interviews with service planners, commissioners, and providers during phase one of the wider study; it was acknowledged that services have become more complex to navigate [11] and an acceptance that reluctance from competing services to work together was due to reduction in funding sources. It is important to recognise that effecting change can be challenging, especially at a time when the National Health Service and local authorities are operating in a climate of significant structural change, combined with the requirements to make major efficiency savings. It is, therefore, vitally important for people with substance misuse issues to establish relationships of trust across whole systems during this difficult economic time [21].

The planning symposium facilitated a co-design of a new integrated model of care, in real time, providing space for participants to work through challenges and agree potential solutions. The idea of the hub and spoke was to reduce the likelihood of disengagement, maximise the availability of appropriate services whilst ensuring it is cost effective [36,37]. Any unwillingness to engage in health and social care services may increase the likelihood of re-referrals. Multiple re-referrals to primary and secondary healthcare as well as third sector organisations are likely to cost far more (in both financial and human terms) than a single successful referral. In recognition of these financial and human costs, it is hoped that the hub and spoke model will support a cost-neutral approach as well as benefitting service users. A hub and spoke approach to healthcare has been utilised by a not-for-profit healthcare provider in Louisiana, USA with great success, providing consistency, increased efficiency, and enhanced quality of care [see 38]. Research has also suggested that the cost of care delivered by a hub and spoke approach is reduced, benefiting patients and society as a whole [39,40].

However, in order for the new proposed model to work in practice, consideration needs to be given to the identification of veterans. Recently, there has been an increase in acceptance that not all veterans identify with the term ‘veteran’. Burdett et al, [33] asked 200 UK ex-forces personnel if they would describe themselves as a veteran. Only 52% said they would, despite being classed as a veteran according to the UK Government. The wording of questions can be a major barrier to identifying and engaging veterans in healthcare services. In the third phase of the larger project this study is a part of, it was suggested that at entry to healthcare services, individuals should be asked “have you ever served in the UK Armed Forces?” rather than “are you a veteran?” which is standard in some healthcare questionnaires [see 26].

Complex presentations are common amongst veteran service users and findings from research concur that this is a typical presentation pattern [e.g. 11,13,25,41]. In the proposed ‘hub and spoke’ model, upon identification of a veteran, a multi-agency support worker would be assigned to the veteran to see them through accessing and engaging in the relevant services. This holistic view centres on empowerment and recognises the pathway as a collective approach, rather than a single service approach. Nonetheless, it was recognised by all of the participants that, the solution to this could be challenging. In response, the proposed use of peer-support workers offers a solution to ensuring consistency throughout veterans’ engagement in services and effective communication across the sectors. Davidson et al, [42] identify that one of the benefits of peer-support for service users is the sense of hope created through meeting people who have overcome difficulties and challenges and are much further along in their recovery. It has also been recognised that peer support workers can help drive forward a recovery-focused, service user-led approach to facilitate better understanding between service providers and users at no extra cost [43].

A limitation of the symposium was a lack of top-level representation from statutory health and social care agencies. In contrast, third sector agencies were the most responsive and had the greatest level of representation at the planning symposium. This is perhaps reflective of the general trend in which third sector organisations in the UK have taken on a growing share of services previously delivered through statutory agencies [44]. In England alone in 2010, over a quarter of charities and social enterprises were active in health and wellbeing, with just under a fifth stating this as their primary focus [45].

Additionally, this hub and spoke model of care was developed within a locality; it is possible that the impact of this model will be limited to the specific geographical location. However, there are elements of this model that could be replicated across the UK following an evaluation of the model in practice.

## Conclusions

Commissioning evidence-based services is key to delivering recovery-focused treatment. To achieve positive change and outcomes, agencies need to work together to identify the needs of veterans, build resilience through a ‘whole systems’ approach and work in partnership. The findings of this study deliver a collaborative co-designed model of care. The ‘hub and spoke’ approach could offer an opportunity to reduce the number of veterans ‘falling through the gaps’ or disengaging from services due to difficulties presented by navigating complex systems. A significant argument in support of this model is that it has been developed from empirical evidence by planners, commissioners, providers and, most importantly, service users.

Following the planning symposium, participants carried forward the ideas presented on the day and the hub and spoke model of care has now been implemented in a local area. To provide a practice-based evidence for the model of care, this will be evaluated over a two-year period to assess the effectiveness of this innovative approach (results available 2021). The complex interaction between ‘old’ ways of working and introducing ‘new’ ways of working will play a key role in the implementation of the new model of care. New initiatives that require change can often fail to deliver. This is acknowledged and will be taken into consideration during the pilot period of the evaluation. The ability to adapt to a new model of care will also require technical changes as well as changes that staff working in services will need to adjust. Participants acknowledged the need to adapt and adjust and identified opportunities to support the role of the peer support worker.
